# Integrative analysis of the microbiome and metabolome in understanding the causes of sugarcane bitterness

**DOI:** 10.1038/s41598-021-85433-w

**Published:** 2021-03-16

**Authors:** Weijuan Huang, Donglei Sun, Lijun Chen, Yuxing An

**Affiliations:** Institute of Bioengineering, Guangdong Academy of Sciences, Guangzhou, 510316 Guangdong China

**Keywords:** Ecology, Microbiology, Molecular biology, Plant sciences

## Abstract

Plant–microbe interactions can modulate the plant metabolome, but there is no information about how different soil microbiomes could affect the sugarcane metabolome and its quality. Here, we collected soil and stalk samples from bitter sugarcane (BS) and sweet sugarcane (SS) to conduct chemical analysis, microbiome and metabolome analysis. Our data revealed lower species diversity in the BS group than in the SS group, and 18 discriminatory OTUs (relative abundance ≥ 0.01%) were identified. Sugarcane metabolomic analysis indicated the different abundances of 247 metabolites between the two groups in which 22 distinct metabolites involved in two flavonoid biosynthesis pathways were revealed. Integrated analysis between soil microbial taxa, stalk chemical components, and soil properties showed that the flavonoid content in stalks and the nitrogen concentration in soil were highly correlated with the soil microbiome composition. Bacteria at the genus level exhibited greater associations with distinct metabolites, and six genera were independently associated with 90.9% of the sugarcane metabolites that play a major metabolic role in sugarcane. In conclusion, this study provided evidences that the interaction between plant–microbiome can change the plant metabolome.

## Introduction

Recent studies have shown that soil microbiomes can impact plant growth and development from the genome to the phenome, as their interactions coevolved and coadapted over time and space^1,2^. Some specific pairwise plant–microbe interactions can modulate a plant’s metabolome, as exemplified by the production of the phytoalexin camalexin in *Arabidopsis* plants infected by pathogens^3^. The effects of soil microbiome-driven changes in leaf metabolomics on the feeding behavior of *Trichopulsia ni larvae* have been determined^4^. Further reports have found evidence of alterations in plant metabolism/traits caused by beneficial microbial colonization or selected microbiomes through stimulated production of phytoalexins and other defense-related compounds^5,6^. Meanwhile, information about how different soil microbiomes could affect a plant’s metabolome is limited.

Sugarcane and tobacco have become the most important economic crops in southern China^7,8^. From our field observation in Guangdong Province, sugarcane with contrasting cropping history (with/without history of tobacco cultivation) turned out to have a contrast taste, where growing of sweet sugarcane in a field with history of tobacco cultivation resulted in bitter taste. However, the underlying causes involved in the occurrence and development of sugarcane bitterness in the field are far from being explored.

Thus, we envisaged this study to comprehensively interrogate soil chemicals, the soil microbiome and sugarcane metabolome to decipher the association of sugarcane bitterness with soil factors and the host metabolites. Here, we applied an integrated approach combining 16S rRNA gene sequencing and liquid chromatography–mass spectrometry (LC–MS) metabolomics to identify microbial diversity and metabolite abundance. Correlation analysis was performed to examine the associations of the soil microbiome, chemicals and the host metabolite profile with sugarcane bitterness.

## Results

### Soil microbial diversity and community structure

**Figure 1:**
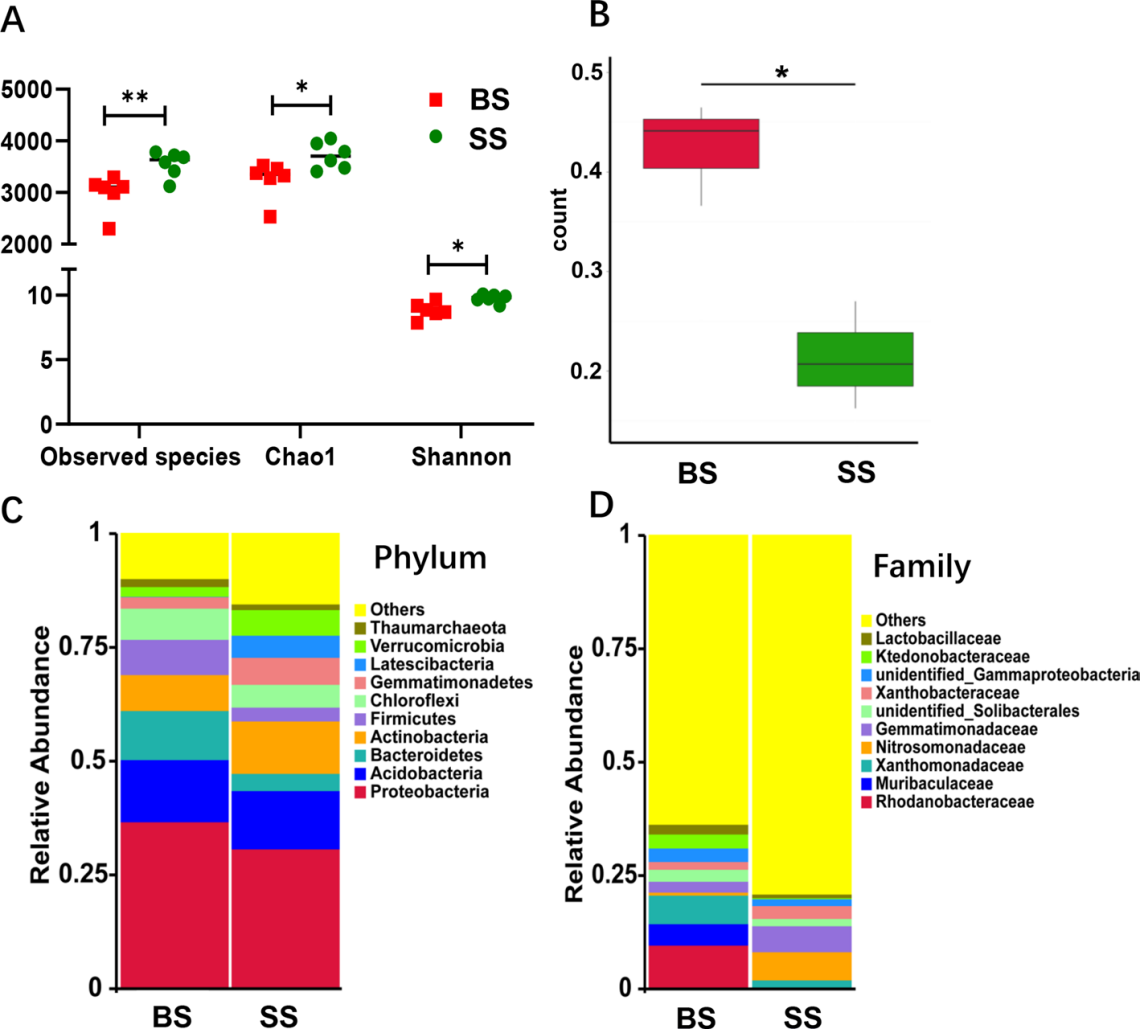
Soil microbiome diversity and structure analysis. *BS*, bitter sugarcane group, *SS*, sweet sugarcane group. (**A**) Alpha diversity differences between the BS and SS groups were estimated by the observed species, Chao1, and Shannon indices. (**B**) Beta diversity differences between the BS and SS groups. Taxonomic differences in microbial community for BS and SS at the phylum (**C**) and family (**D**) levels. **P < 0.01, *P < 0.05. Figures were produced using R (v3.4.3, https://www.R-project.org).

In total, 1,334,381 reads were obtained for the bacterial 16S rRNA genes by high-throughput sequencing. After screening these gene sequences with strict criteria (described in “Materials and methods”), 1,061,916 valid sequences were obtained, accounting for 79.6% of the raw reads. Figure 1A shows that the observed richness, Chao1, and Shannon index in the SS (sweet sugarcane) group supported significantly higher richness (*P* < 0.01) and diversity (*P* < 0.05) than in the BS (bitter sugarcane) group. It suggested that BS and SS differed regarding several metrics considered in alpha-diversity. Beta-diversity analysis of the two groups showed that the microbial community structure of BS was significantly different from that of SS (*P* < 0.05), i.e., a distinct separation was observed (Fig. 1B, Fig. S1).

**Figure 2:**
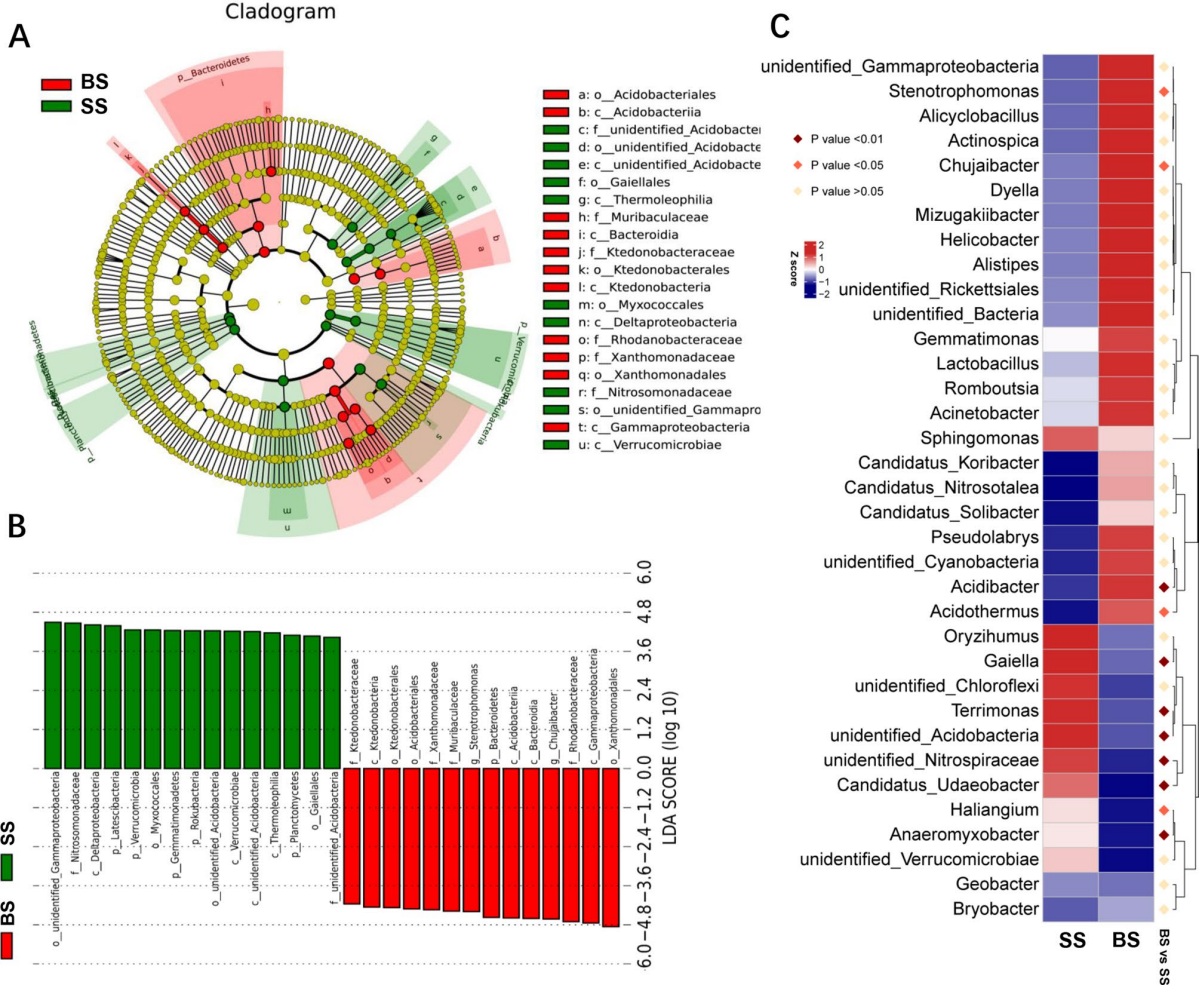
Identification of compositional differences for bacterial species in the BS and SS groups. (**A**) Cladogram indicating the phylogenetic distribution of microbiota correlated with the two groups. (**B**) Linear discriminant analysis (LDA) integrated with effect size (LEfSe), reflecting the differences in abundance between the two groups. (**C**) Heatmap of the relative abundances of the top 30 bacterial genera that differentiate the BS and SS groups. OTUs are shown from lower abundance (in blue) to higher abundance (in red) for the z-transformed data. Data were analyzed by the Wilcoxon rank-sum test (Mann–Whitney U test). Figures were produced using R (v3.4.3, https://www.R-project.org).

A phylum-level analysis demonstrated the compositional differences of soil microbial communities, showing that all samples were dominated by Proteobacteria, Acidobacteria, Bacteroidetes, Actinobacteria, Firmicutes, Chloroflexi, Gemmatimonadetes, Latescibacteria, and Verrucomicrobia (Fig. 1C). With respect to the top 10 phyla, the soil bacterial community of the BS group had greater abundances of Bacteroidetes, Firmicutes, and Chloroflexi (*P* < 0.05), compared with lower abundances of Actinobacteria, Latescibacteria, and Verrucomicrobia relative to the SS group (*P* < 0.05). In the analysis of the microbiota composition at the family level, Rhodanobacteraceae, Xanthomonadaceae, and Ktedonobacteraceae were highly enriched in BS compared to SS (*P* < 0.05), while Nitrosomonadaceae and Gemmatimonadaceae were highly enriched in SS versus BS (*P* < 0.05) (Fig. 1D). In order to distinguish the predominant taxa, LEfSe [linear discriminant analysis (LDA) integrated with effect size] was used to generate a cladogram to identify the specific bacteria associated with the BS group (Fig. 2A,B). We identified 81 distinctive genera from the soil bacterial community of the two groups (Table S1). Some bacteria, including Gammaproteobacteria, Acidobacteriia, Ktedonobacteraceae, Muribaculaceae, and Stenotrophomonas, were all significantly overrepresented (all LDA scores (log_10_) > 4) in the bitter sugarcane group of BS, whereas Deltaproteobacteria, Verrucomicrobiae, Thermoleophilia, and Nitrosomonadaceae were the most abundant microbiota in the control group (SS; LDA scores (log_10_) > 4). Among them, the top 18 distinct genera (*P* < 0.05, relative abundance ≥ 0.01%) were selected as key discriminants (Fig. 2C, Table S2). In particular, the genus *Chujaibacter* in Rhodanobacteraceae and *Stenotrophomonas* in Xanthomonadaceae were highly abundant in BS versus SS (*P* < 0.05).

At the OTU level, Venn diagrams were constructed to better represent the results of shared richness between microbial samples from two sites, which, according to a detailed analysis, represented the number of OTUs shared between the two groups (Fig. S2). The two soil groups had a high number of shared sequences (3173 OTUs, 63.1%), revealing the presence of a strong core microbiota composed of Proteobacteria (27.4%), Actinobacteria (9.9%), Acidobacteria (9.7%), and Bacteroidetes (8.9%) (Fig. S2, Table S3). Bacterial functional profiles were inferred from 16S rRNA gene data using PICRUSt. Differences in the microbial function of the soil microbiome between the two groups were compared (Fig. S3). Compared to the SS group, the BS group exhibited lower metabolism of carbon fixation, citrate cycle, and chlorophyll synthesis (*P* < 0.05).

In addition, the relative abundance of soil nitrogen-fixing bacteria in the two groups was compared. Figure S4 shows that *Nitrospira japonica* was significantly enriched in SS compared with BS (*P* < 0.05), indicating that the soil in SS could have a higher efficiency of nitrogen conversion and metabolism versus BS. The bacterial nitrogen-fixing-associated gene *NifH* was further detected and showed a greater abundance in SS compared to BS (Fig. S4).

### Metabolomics analysis of the sugarcane stalks

**Figure 3:**
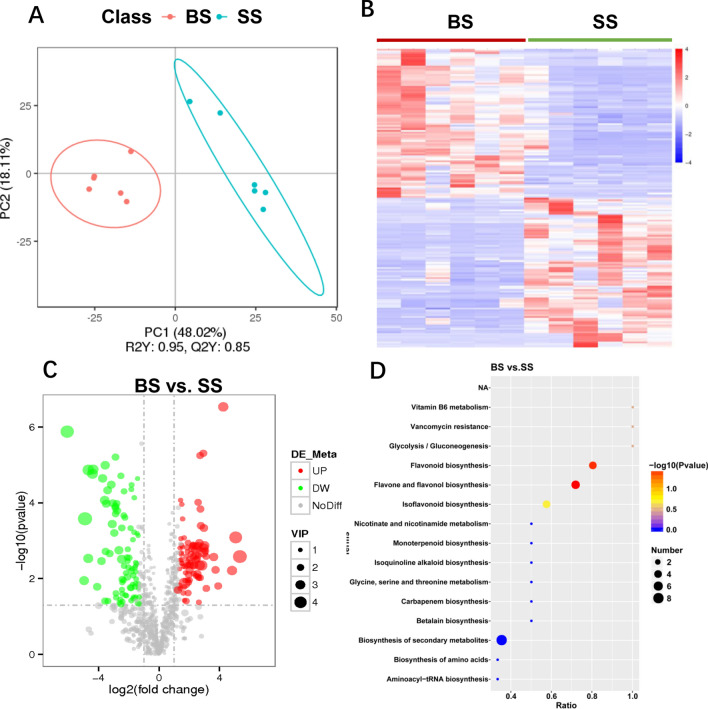
Important discriminatory metabolites identified by clustering, correlation and multivariate analysis between the BS and SS groups. (**A**) PLS-DA analysis displaying the grouped discrimination of the BS and SS groups by the first two PCs. (**B**) Hierarchical clustering analysis (HCA) for the BS and SS metabolites based on their z-normalized abundances. (**C**) Variable Importance in Projection (VIP) scores of the important discriminatory metabolites. (**D**) Scatter plot of top 15 KEGG pathway enrichment for differential metabolites in the BS and SS groups. Panel A was produced using metaX (v1, https://github.com/wenbostar/metaX), and panel A–D were produced using R (v3.4.3, https://www.R-project.org).

A multivariate analysis method, PLS-DA, was used to identify the key compounds (detected by LC–MS) responsible for the differentiation. Score plots based on the first two components showed that the two groups were well separated (Fig. 3A). In total, we detected 1245 metabolites in stalk tissues based on LC–MS analyses. Among these metabolites, 247 (19.8%) were differentially abundant between sugarcane stalks collected from site A (BS) and from site C (SS) (Fig. 3B, *P* < 0.05; Table S4). These metabolites, including amino acids, organic acids, sugars, and sugar alcohols, fatty acids, lipids, benzenoids, phenylpropanoids, and polyketides, are involved in multiple biochemical processes in sugarcane stalks.

To identify the compounds responsible for the difference, the VIP combined with the univariate statistical results (Table S4) was used to select variables with the most significant contribution to the discrimination of the sugarcane compounds of the two groups. Statistically significant differences were observed between the BS and SS groups shown in the volcano map (Fig. 3C). Compared to SS, 112 metabolites were significantly increased in BS, and 135 metabolites were significantly decreased (VIP > 1, *P* < 0.05). Among them, lamioside (62.94-fold increase), primisulfuron (57.93-fold increase), 3,5-dimethylpyrazin-2-ol (40.66-fold increase), sitaxentan (33.53-fold increase), crolibulin (26.50-fold increase), 2-formylpyridine (7.09-fold increase), (z)-desulfoglucotropeolin (6.45-fold increase), and asparagine (3.23-fold increase) were produced in greater concentrations in BS, while leucoside (67.59-fold decrease), isovitexin 2″-*O*-arabinoside (29.84-fold decrease), vaccarin (25.65-fold decrease), kuromanin (21.22-fold decrease), and robinin (20.61-fold decrease) were greatly reduced in BS relative to SS (VIP > 3, *P* < 0.05).

Through KEGG pathway annotation, 194 metabolites of these 247 distinct metabolites were mapped onto 13 different KEGG metabolic pathways, including amino acid metabolism, metabolism of cofactors and vitamins, metabolism of terpenoids and polyketides, and biosynthesis of other secondary metabolites. However, the KEGG-enrichment scatterplot showed that there were only two distinguishing pathways including flavonoid biosynthesis, flavone and flavonol biosynthesis which were significantly enriched in BS compared to SS (*P* < 0.05; Fig. 3D). Therefore, 22 distinct metabolites were selected from these two KEGG pathways for correlation analysis, including vitexin, naringin, apigenin, hesperetin, rhoifolin, and chlorogenic acid (Table 1).

**Table 1:** Metabolomic analysis of the sugarcane stalks in BS compared to SS.

Metabolite	FC	P value	VIP	Changes (up/down)
Valine	8.64	0.0026	1.98	Up
dl-Arginine	4.77	0.0013	1.72	Up
l-Pyroglutamic acid	4.49	0.0016	1.76	Up
d-Serine	3.46	0.0391	1.76	Up
Asparagine	3.23	0.0132	3.57	Up
7-Hydroxycoumarine	3.04	0.0048	1.14	Up
5-Aminovaleric acid	9.94	0.0008	2.33	Up
Chlorogenic acid	15.26	0.0013	2.37	Up
Glycine anhydride	2.77	0.0146	2.45	Up
Luteolin	0.38	0.0449	1.61	Down
Neodiosmin	0.33	0.0327	1.17	Down
Trigonelline	0.32	0.0006	1.28	Down
Phloroglucinol	0.30	0.0142	1.16	Down
Apigenin	0.28	0.0178	1.67	Down
Hesperetin	0.23	0.0028	1.63	Down
Biochanin A	0.20	0.0001	1.60	Down
4-Methoxycinnamaldehyde	0.19	0.0126	1.43	Down
Rhoifolin	0.13	0.0001	2.02	Down
Vitexin	0.10	0.0022	2.10	Down
Hesperidin	0.08	0.0001	2.73	Down
Kuromanin	0.05	0.0000	3.19	Down
Naringin	0.12	0.0010	2.06	Down

*BS* bitter sugarcane group, *SS* sweet sugarcane group, *FC* fold change, *VIP* variable importance in projection.

### Correlation between soil properties, the soil microbiome, and the sugarcane metabolome

**Figure 4:**
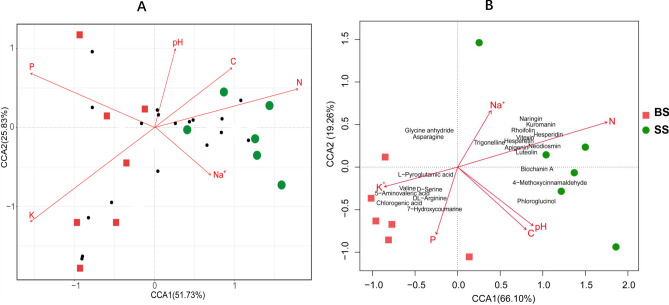
CCA analysis of soil properties correlated with the soil microbial communities and metabolites of sugarcane. (**A**) Correlation between the content of soil properties and the relative abundance of the top 18 soil differential microbes at the genera level (black dot corresponds to a bacterial genus). (**B**) Correlation between the content of soil properties and 22 distinct metabolites of sugarcane stalk. Vectors show the relationship between the secondary variables and the microbial community data, where the length and angle are determined by the correlation of each variable with the ordination axes. ‘pH’, ‘C’, ‘N’, ‘P’, ‘K’, and ‘Na’ refer to soil properties. Figures were produced using R (v3.4.3, https://www.R-project.org).

Analysis showed that the soil from the BS group only had a significantly lower available nitrogen content and significantly higher potassium ion content compared to soil from the SS group (*P* < 0.05; Table 2). The contents of total carbon, PO_4_-P, and other elements did not show dramatic changes. Canonical correlation analysis (CCA) based on the genus-specific abundances (Table S2) from each soil sample was used to determine how the various soil microbial communities correlated with soil edaphic factors. The bacterial community of BS was positively correlated with P and K, but negatively correlated with C, N, pH, and Na^+^ (Fig. 4A). On the contrary, the correlation result was opposite for the bacterial community of SS. Among the 18 distinct bacterial genera associated with soil properties in Fig. 4A, we found that nitrogen had a significantly positive correlation (*P* < 0.05) with *Reyranella* (*r* > 0.99), *Dongia* (*r* > 0.94), *Gaiella* (*r* > 0.92), *Candidatus_Udaeobacter* (*r* > 0.91), and *unidentified_Acidobacteria* (*r* > 0.88), and had a significantly negative correlation (*P* < 0.05) with *Ralstonia* (*r* < − 0.83). On the contrary, potassium was found negatively correlated (*P* < 0.05) with *Candidatus_Udaeobacter* (*r* < − 0.82) and *Reyranella* (*r* < − 0.81), and positively correlated (*P* < 0.05) with *Ralstonia* (*r* > 0.84) (Table S5).

**Table 2:** Origin and characteristics analysis of field soils used in this study.

Name	Location	pH	C (g/kg)	N (mg/kg)	PO_4_-P (mg/kg)	K (mg/kg)	Na^+^ (mg/kg)	Cl^−^ (mg/kg)	1/2Mg^2+^ (cmol/kg)	CEC (cmol/kg)
BS	Shifeng Village, Jiangling County, Guangdong, China	5.53	12.23	78.21	18.89	74.24	0.36	8.65	0.98	6.07
SS	Zhangkeng Village, Jiangling County, Guangdong, China	5.87	18.15	135.41*	4.69	27.28	0.40	7.23	0.94	6.80

*p < 0.05.

To determine the impact of soil chemicals on the sugarcane metabolome, CCA was performed and heatmaps (Fig. 4B, Fig. S5) constructed using the Pearson’s correlation coefficient to determine the correlations between the distinct sugarcane metabolites and soil edaphic factors. The correlation analysis revealed that only certain soil elements, including nitrogen and potassium in particular, had significant correlations with the distinct metabolites (Fig. 4B). Nitrogen was significantly positively associated (*P* < 0.05) with hesperetin (*r* > 0.86), luteolin (*r* > 0.82), and neodiosmin (*r* > 0.84), and significantly negatively correlated (*P* < 0.05) with dl-arginine (*r* < − 0.94), 7-hydroxycoumarine (*r* < − 0.81) and l-pyroglutamic acid (*r* < − 0.81). Potassium was positively correlated (*P* < 0.05) with chlorogenic acid (*r* > 0.86) and dl-arginine (*r* > 0.83) (Table S6).

### Soil microbiome strongly linked to the sugarcane metabolites

**Figure 5:**
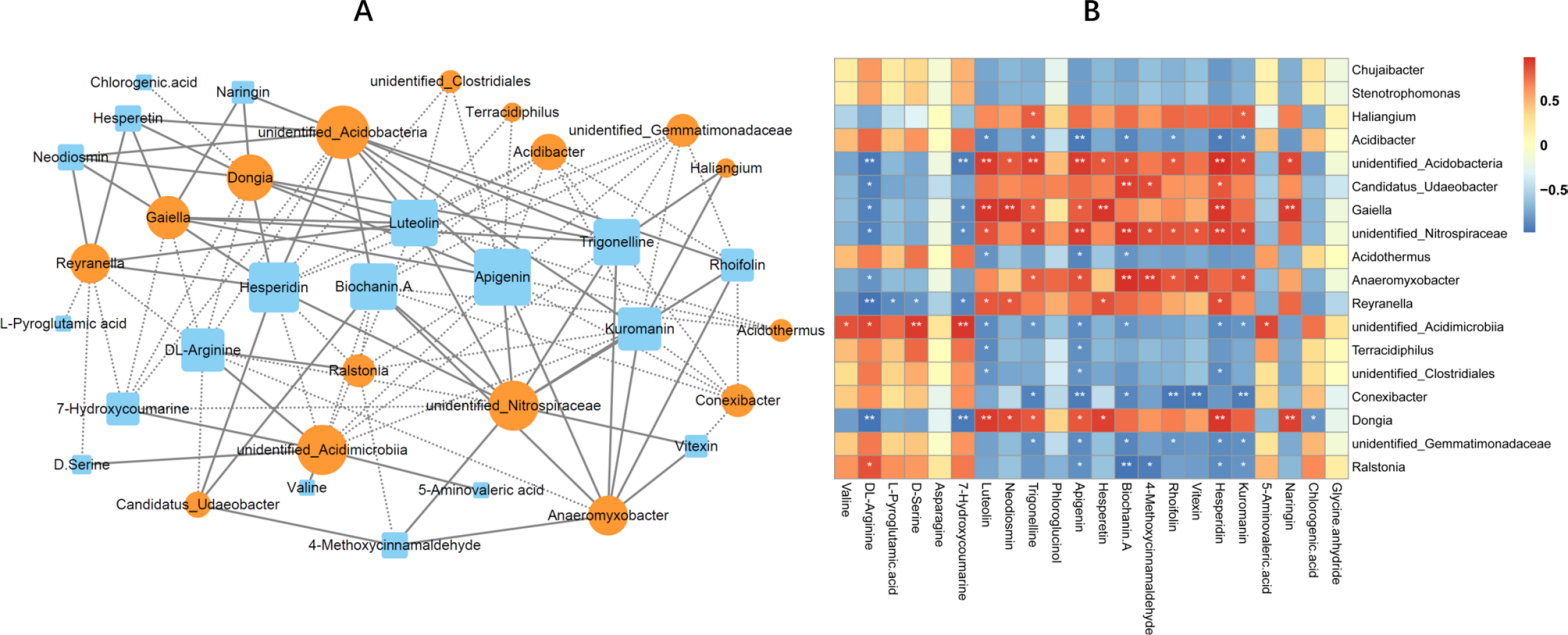
Integrated correlation-based network analysis of the soil microbes and sugarcane metabolites. (**A**) Correlation-based network analysis (Pearson’s correlation) of microbes and metabolites (solid line and dotted line representing positive correlation and negative correlation, respectively). (**B**) Pearson’s correlation analysis from the entire network between 18 differential microbes and 22 distinct metabolites showed in the heatmap (positive correlation in red, negative correlation in blue; *P < 0.05, **P < 0.01). Figures were produced using R (v3.4.3, https://www.R-project.org).

To explore the functional correlation between the soil microbiome changes and metabolite perturbations, a Pearson’s correlation matrix was generated by calculating the Pearson’s correlation coefficient between the different bacterial abundances (18 genera) and distinct metabolites (22 compounds mainly involved in the flavonoid biosynthesis pathway, VIP > 1.0) of the BS and SS groups (Table S7). Significant associations were selected (*P* < 0.05), and their biological networks were visualized using Cytoscape v3.7.1, based upon which clear correlations were identified between the perturbed soil microbiome and altered metabolite profiles (Fig. 5). As expected, the composition of the soil microbiome was widely associated with the sugarcane metabolic content. At a 5% false discovery rate (FDR), we found 108 associations between sugarcane metabolites and microbial species (Fig. 5A). In particular, 88.9% of the species were associated with 86.4% of the sugarcane metabolites. We observed 50.9% positive and 49.1% negative associations with microbial species and metabolites, respectively. On average, each metabolite was associated with five species. Six microbial species played a major metabolic role and were independently associated with 90.9% of the sugarcane metabolites: *unidentified_Acidobacteria* (12 metabolites), *unidentified_Nitrospiraceae* (11 metabolites), *unidentified_ Acidimicrobiia* (11 metabolites), *Dongia* (10 metabolites), *Galiella* (9 metabolites), *Reyranella* (8 metabolites), and *Anaeromyxobacter* (8 metabolites).

Among the distinct metabolites, apigenin, biochanin A, hesperidin, luteolin, and trigonelline had ten correlations on average with different microbial species. Examining these metabolites, we noted that apigenin had the most associations including five positive correlations and eight negative correlations. The down-regulation of apigenin (0.28-fold) was highly positively correlated (*P* < 0.05) with the accumulation of *unidentified_Nitrospiraceae* (*r* > 0.95), *unidentified_Acidobacteria* (*r* > 0.94), *Anaeromyxobacter* (*r* > 0.87), *Dongia* (*r* > 0.84), and *Gaiella* (*r* > 0.83) and was negatively correlated with the accumulation of *Acidibacter* (*r* < − 0.93), *Conexibacter* (*r* < − 0.92), and the remaining six genera (Fig. 5B). Chlorogenic acid (15.26-fold change, the most up-regulated one among the 22 distinct metabolites) was negatively associated (*P* < 0.05) only with *Dongia* (*r* < − 0.81). Kuromanin (0.05-fold change, the most down-regulated metabolite) was positively associated (*P* < 0.05) with *unidentified_Nitrospiraceae* (*r* > 0.91) and *unidentified_Acidobacteria* (*r* > 0.90) and was negatively associated (*P* < 0.05) with *Conexibacter* (*r* < − 0.92) and *Acidibacter* (*r* < − 0.86). Thus, the soil microbiome strongly linked to the sugarcane metabolites may contribute to the different flavors of the two groups.

## Discussion

The quality of crops, especially the edible quality, e.g., of sugarcane, is of great significance to the economic value of agricultural products, as well as to the safety of food health^9^. Soil microorganisms that interact with host plants are known to stimulate the production of a rich and different repertoire of metabolites in plants^10^. However, the relationship between the below-ground microbiome and above-ground plant quality remains unclear. In this study, we integrated the metabolomics data from sugarcane stalks with 16S amplicon sequence data derived from soil microbiota to investigate the causes of bitter sugarcane grown in the soil with preceding cultivation of tobacco. The content of sucrose in both the BS group (bitter sugarcane) and the SS group (sweet sugarcane) was first tested and showed no differences. Furthermore, the nontargeted metabolomics approach for the comprehensive analysis of bitter-related compounds in sugarcane stalks of the BS group detected many differential metabolic signatures compared with the SS group. In light of our results, we identified a larger number of decreasing metabolites (including luteolin, hesperidin, naringin, and vitexin) than increasing metabolites (chlorogenic acid, 7-hydroxycoumarine, and amino acids including valine and D-serine) in the BS group (Table 1, Table S4), which could be used as bitterness-derived metabolic biomarkers for sugarcane. However, the flavonoid biosynthesis was highly enriched in the BS group compared to the SS group (*P* < 0.05). Many (iso)flavonoids have an undesirable bitter taste but are associated with beneficial health effects^11^. Thus, whether these distinct metabolites revealed in the bitter sugarcane will bring health benefits to consumers still needs further investigation. Additionally, metabolites such as trehalose and raffinose were reported to show positive correlations with sucrose concentration^12^. However, these two metabolites detected in the sugarcane stalks of the two groups did not differ significantly.

Furthermore, our study revealed that soil properties and the soil microbiome were highly associated with the metabolic changes in sugarcane stalks. In particular, significant correlations were found between the soil microbiome and sugarcane metabolites based on co-occurrence network analysis. Many bacterial genera with significant differences in the relative abundance of the two groups were highly associated with distinct metabolites involved in the biosynthesis of flavonoids. For instance, unidentified_*Acidobacteria*, *Gaiella*, unidentified_*Nitrospiraceae*, and *Dongia* with high abundances in SS versus BS had significantly positive associations with luteolin, trigonelline, apigenin, biochanin A, and hesperidin which were greatly enriched in SS versus BS, while *Acidibacter*, unidentified_*Acidimicrobiia*, and unidentified_*Gemmatimonadaceae* were negatively associated with these metabolites. This suggested that these bacterial groups may have the potential to influence sugarcane secondary metabolites, resulting in a different flavor. This finding is in accordance with previous studies that the soil microbiome is involved in the synthesis of important flavor compounds that improve the fruit quality or flavor of grapes and strawberries^13,14^. Because soil is considered the main source of plant-associated bacterial colonizers, more than 90% of all OTUs inhabiting the roots, stalks, and leaves of sugarcane are reported to be present in the soil microbiome^15^. Therefore, changes in the structure of the soil microbiome can largely affect the sugarcane stalk-associated microbial communities linked to the modulation of stalk metabolites. Some plant diseases related to microbes such as orange affected by Huanglongbing or citrus greening disease were found associated with a bitter off-flavor of orange juice compared to healthy oranges^16^. Likewise, sugarcane smut disease was reported to have negative effects on sucrose accumulation and juice quality^17^. Nonetheless, all the collected sugarcane samples in this study were healthy, and diseased sugarcane samples above the ground were barely detected in the field during our survey, which can therefore be excluded from our considerations of the relevant causes of sugarcane bitterness.

Soil types have been revealed to effectively modulate bacterial communities more than geographical distance^18^. In our study, some phyla showed particular correlations with soil nutrients, including the positive correlation of Firmicutes with K and P, and of Gemmatimonadetes and Nitrospirae with total carbon and nitrogen. The type and quantity of nitrogen fertilizer were reported to affect the physical, chemical, and biochemical properties of soil, as well as soil microbial communities^19^. We found that only nitrogen was significantly enriched in SS, which had a high abundance of nitrogen-fixing bacteria, and was positively associated with many bacteria including *Reyranella*, *Dongia*, *Gaiella*, *Candidatus_Udaeobacter*, and *unidentified_Acidobacteria.* However, the abundance of these bacteria was much lower in BS, which may reduce the soil capacity to fix nitrogen and thus influence the ability of the plants to synthesize nitrogen-related compounds. In addition, nitrogen nutrition was found to have significant effects on the plant metabolome that could impact amino acids, carbohydrates, and secondary metabolites^20,21^. Our study revealed that the low nitrogen in BS was positively correlated with 7-hydroxycoumarine, dl-arginine, and l-pyroglutamic acid but negatively correlated with the accumulation of secondary metabolites in sugarcane including hesperidin, neodiosmin, and luteolin, implying that the nitrogen-induced effects may be mediated by soil microorganisms. In summary, manipulating the soil microbiome associated with the biogeochemical cycling of nitrogen would be of great significance to nitrogen management for improving plant quality in agricultural production.

We observed that the content of nitrogen in the soil of BS with the preceding cultivation of tobacco was significantly decreased while P and K were enriched compared to SS in which maize, potato, and rice were previously grown. This is in accordance with the findings of previous reports that tobacco production can modulate the characteristics of soil nutrients, resulting in an insufficiency of N, Zn, and Mn, while P and K cannot be fully utilized in soil^22,23^. Due to the differences in the biological characteristics of the crop itself and the cultivation techniques, agricultural production will have discriminatory effects on soil, including physical and chemical properties, nutrients, and the water contents, with subsequent effects on the succeeding crops^24–26^. Therefore, the effects of crop biomass, soil N availability, and nutrient absorption and residues of N, P, and K after the cultivation of different preceding crops could be significantly divergent^27,28^. These influences would further impact the structure and function of the soil microbiome, as we observed that nitrogen-fixing bacteria and the associated *NifH* gene were significantly decreased in the BS group compared to the SS group. Collectively, our results suggest that soil properties and the soil microbiome were highly associated with the changes in sugarcane metabolites that may affect the sugarcane metabolome directly or indirectly, which could result in the bitterness of sugarcane due to the enrichment of flavonoid biosynthesis observed in the BS group. This finding should be a key consideration in future plant–microbial interactions research attempting to integrate plant metabolomes and microbiomes to understand the links between microbial communities and plant quality.

In conclusion, this study investigated the changes in the microbial community in soils and metabolomics variations of sugarcane stalks as well as soil properties to reveal the causes of sugarcane bitterness. Using a nontargeted LC–MS-based metabolomics approach, we observed different metabolites were associated with the two types of sugarcane. Many significant correlations were revealed between the soil microbiome and sugarcane metabolites based on co-occurrence network analysis. Our results suggest that the soil microbiome has a strong impact on the edible quality of sugarcane by modulating its secondary metabolites.

## Materials and methods

### Sample collection and processing

Samples of sugarcane stalks and rhizosphere soil (six replicates for each type of material, three or more pooled samples per replicate) were collected randomly in February, 2019 from two different villages, Shifeng (N 25° 51′ 11″, E 116° 9′ 40″) and Zhangkeng (N 24° 41′ 23″, E 116° 10′ 40″) with a distance of less than 10 km, both located in Jiaoling County, Guangdong Province, China. Samples of bitter sugarcane collected from Shifeng village were labeled as BS (voucher specimens no. SCB001-SCB024), while those sweet sugarcane from Zhangkeng village were labeled as SS (SCS001–SCS018). All specimens were identified by prof. Yuxing An, and deposited in Guangzhou Sugarcane Industry Research Institute, Guangdong, China. Three subsamples of sugarcane stalks (including the basal, middle and top section) in one site were collected and pooled into one experimental unit, then put into Ziplock bags with ice, and transported to the laboratory for further processing. Soil sampling was limited to the top 15 cm depth and collected from at least six locations per soil/plant site. Three or more samples were pooled, and subsamples for nutrient (500 g) and microbial community (5 g) analyses were stored at − 80 °C until analysis. Soil samples collected from both sites belong to red soil, and were analyzed for pH, total carbon (C), available nitrogen (N), PO4-P, K^+^, Na^+^, Cl^−^, 1/2Mg^2+^, and cation exchange capacity (CEC) at the Soil Testing Company of Nanjing (Nanjing, China).

### DNA extraction and 16S rRNA gene amplicon sequencing

Microbial DNA from soil samples was extracted using a Soil Microbe DNA Kit (QIAGEN, USA). All DNA extractions were quantified spectrophotometrically and diluted to a final concentration of 10 ng/μL. The V4 hypervariable region of the 16S rRNA gene was amplified for each sample using barcoded universal primers (515F: 5′-GTGCCAGCMGCCGCGGTAA-3′; 806R: 5′-GGACTACHVGGGTWTCTAAT-3′)^29^. PCR reactions were carried out in 30 µL reactions with 15 µL of Phusion High-Fidelity PCR Master Mix (New England Biolabs, USA), 0.2 µM of forward and reverse primers, and about 10 ng of template DNA. Thermal cycling consisted of initial denaturation at 98 °C for 1 min, followed by 30 cycles of denaturation at 98 °C for 10 s, annealing at 50 °C for 30 s, elongation at 72 °C for 30 s, and finally 72 °C for 5 min. PCR products were subsequently subjected to electrophoresis on a 2% agarose gel, stained with ethidium bromide, and the targeted fragment size was purified with an AxyPrepDNA gel extraction kit (Axygen, China). Sequencing libraries were generated using Ion Plus Fragment Library Kit 48 rxns (Thermo Scientific, USA), following the manufacturer’s recommendations. The library quality was assessed on a QuantiFluor-ST fluorometer (Promega, USA) and Agilent 2100 Bioanalyzer system (Agilent, USA). Lastly, the library was sequenced on an Ion S5 XL platform, and 400 bp/600 bp single-end reads were generated. All high-throughput sequencing was completed by Novogene Technology Co., Ltd. (Beijing, China).

The raw sequences were processed using QIIME (version 1.9.1, http://qiime.org)^30^. Adaptors and primers were removed using AdapterRemoval v2^31^. Reads were merged and filtered by size (according to the primer set) and quality (Phred quality score > 20). The sequences were then clustered into operational taxonomic units (OTUs) using an open reference strategy based on 97% identity with the GreenGenes Database v13_5^32^ as the reference. Taxonomy was assigned with an RDP classifier^33^ retrained with SILVA (release 115, http://www.arb-silva.de) for the bacterial 16S rRNA gene database. Chimeric OTUs were identified using uchime (version 4.2, http://drive5.com/usearch/manual/uchime_algo.html) and removed from the OTU table.

### Metabolomic profiling

Metabolites of sugarcane stalks were analyzed by using a non-targeted LC–MS based metabolomics analysis. In brief, approximately 100–200 mg of fine stalk tissue of sugarcane was ground, and the homogenate was resuspended with prechilled 80% methanol and 0.1% formic acid, and 1 mL of the extract was dried in liquid nitrogen^34^. The mobile phase A was 0.1% formic acid in water for positive, and 5 mmol/L ammonium acetate in water for negative, and the mobile phase B was acetonitrile. The elution gradient was set as follows: 0 min, 1% B; 1 min, 1% B; 8 min, 99% B; 10 min, 99% B; 10.1 min, 1% B; 12 min, 1% B. The flow rate was 0.5 mL/min. The injection volume was 2 μL. The raw data files generated by UHPLC-MS/MS were processed using Compound Discoverer 3.0 (CD3.0, Thermo Fisher, USA) to perform peak alignment, peak picking, and quantitation for each metabolite. The normalized data were used to predict the molecular formula based on additive ions, molecular ion peaks and fragment ions. Then, peaks were matched with the mzCloud (https://www.mzcloud.org/) and ChemSpider (http://www.chemspider.com/) database to obtain the accurate qualitative and relative quantitative results.

These metabolites were annotated using the KEGG database (http://www.genome.jp/kegg/), HMDB database (http://www.hmdb.ca/), and Lipidmaps database (http://www.lipidmaps.org/). Principal component analysis (PCA) and partial least squares discriminant analysis (PLS‐DA) were performed using metaX software^35^. We applied univariate analysis (Student’s t test) to calculate the statistical significance (*P* value). The metabolites with VIP (variable importance in projection) > 1, *P* value < 0.05, and fold change (FC) ≥ 2 or FC ≤ 0.5 were considered to be differential metabolites. Volcano plots were used to filter metabolites of interest which based on the log_2_(FC) and − log_10_(*P* value) of the metabolites. For clustering heat maps, the data were normalized using z scores of the intensity areas of differential metabolites, and the maps were drawn using R’s pheatmap package. The functions of these metabolites and metabolic pathways were studied using the KEGG database.

### Statistical analysis

Statistical analyses of bacterial 16S rRNA gene sequences were carried out in R^36^. To characterize the richness in a specific rhizosphere community, custom R scripts were used to obtain Shannon–Wiener curves, Venn diagrams, and the microbial community bar plots. In the β-diversity analyses, the R package vegan^37^ was used to obtain the heatmap. Phylogenetic Investigation of Communities by Reconstruction of Unobserved States (PICRUSt) was used to predict the metabolic function of the microbial community including nitrogen, methane, and energy metabolisms^38^. Significant differences in the microbial communities between two groups were analyzed using Student’s t test and the Wilcoxon test. For metabolites, statistical analyses were performed using Python, version 2.7.3 (https://www.python.org/downloads/release/python-273/) and CentOS (release 6.6, https://www.centos.org). When data were not normally distributed, normal transformations were attempted using an area normalization method^39^. All analyses were performed using R software program (version 3.4.3, https://cran.R-project.org),

### Co-occurrence network analysis

Significant associations were calculated using Pearson’s correlation coefficient and were visualized using Cytoscape v3.7.1 (https://cytoscape.org) using an edge-weighted spring-embedded layout^40^. Hub microorganisms, i.e., highly interconnected OTUs in the microbial networks^41,42^ were identified as those OTUs (nodes) in each network that were more central, based on selected cut-off values for the degree (number of neighbors/correlations in the network), betweenness centrality (fraction of shortest paths a node is on), and closeness centrality (1/[(distance to all other nodes]), as calculated in Cytoscape v3.7.1.

### Ethical statements

We confirm that the use of plant parts in the present study complies with the Convention on Biological Diversity. *Saccharum officinarum* plant stalks were used in this study. Sweet sugarcane (SS) and bitter sugarcane (BS) selected from Zhangkeng village and Shifeng village (Jiaoling County, Guangdong Province, China), respectively, were kindly provided by Jiaoling Agriculture Bureau (Jiaoling County, Guangdong Province, China).

## Supplementary Information

Supplementary Information 1.

Supplementary Information 2.

## Data Availability

Raw 16S rRNA amplicon-seq was submitted to SRA NCBI with submission no. PRJNA561066. LC–MS raw data were submitted to MetaboLights with submission no. MTBLS1689.
