# Supplementary material for: Integrative analysis of the microbiome and metabolome in understanding the causes of sugarcane bitterness
Source: Sci Rep. 2021 Mar 16;11:6024. doi: 10.1038/s41598-021-85433-w (PMC7966368; doi:10.1038/s41598-021-85433-w)
Supplement: Supplementary file 1 — Supplementary Information 1. [file 41598_2021_85433_MOESM1_ESM.pdf]

Supplementary Information

Article Title: Integrative analysis of the microbiome and metabolome in understanding the causes of sugarcane bitterness

Authors' names: Weijuan Huang, Donglei Sun, Lijun Chen, Yuxing An*

***Corresponding author:** Yuxing An, Department of Crop Stress Management, Institute of Bioengineering, Guangdong Academy of Sciences, No.10 Shiliugang Road, Haizhu District, Guangzhou 510316, Guangdong, China

E-mail: **anyuxing@hotmail.com**

Supplementary Figures

Fig. S1 Weighted UniFrac PCoA plots
Fig. S2 Identification of shared OTUs of the BS and SS groups
Fig. S3 KEGG function prediction
Fig. S4 Relative abundance of nitrogen fixing bacteria and associated gene
Fig. S5 Correlation analysis between soil properties and distinct metabolites of sugarcane

Supplementary Tables

Table S1. Identification of 81 distinctive genera from soil bacterial community in the BS and SS groups.
Table S2. Relative abundaces of 18 discriminatory genera as key discriminants.
Table S3. OTU distribution of each group.
Table S4. Identification of differential sugarcane metabolites between the BS and SS groups.
Table S5. Correlations between soil properties and soil microbiome.
Table S6. Correlations between soil properties and sugarcane metabolites.
Table S7. Correlations between soil microbiome and sugarcane metabolites.

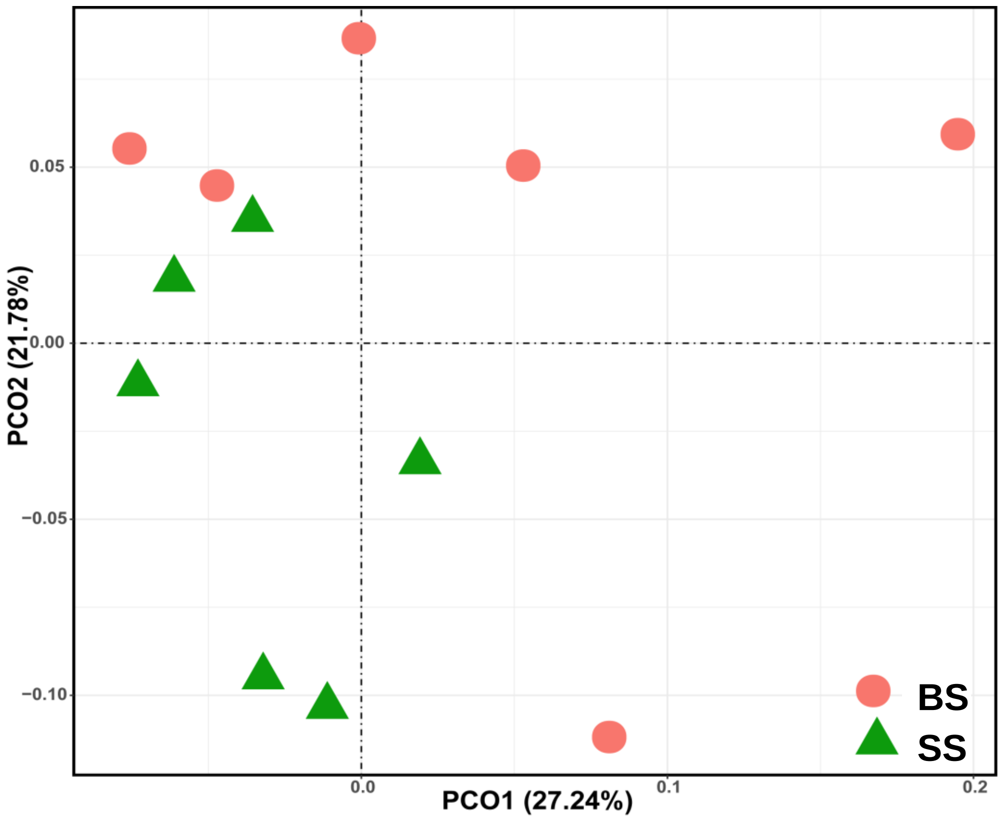

Fig. S1 Weighted UniFrac PCoA plots.
PCoA plot base of the relative abundance of OTUs (97% similarity level) showing bacterial structural clustering. BS group (red dots), bitter sugarcane; SS group (green dots), sweet sugarcane, where dots represent individual samples.

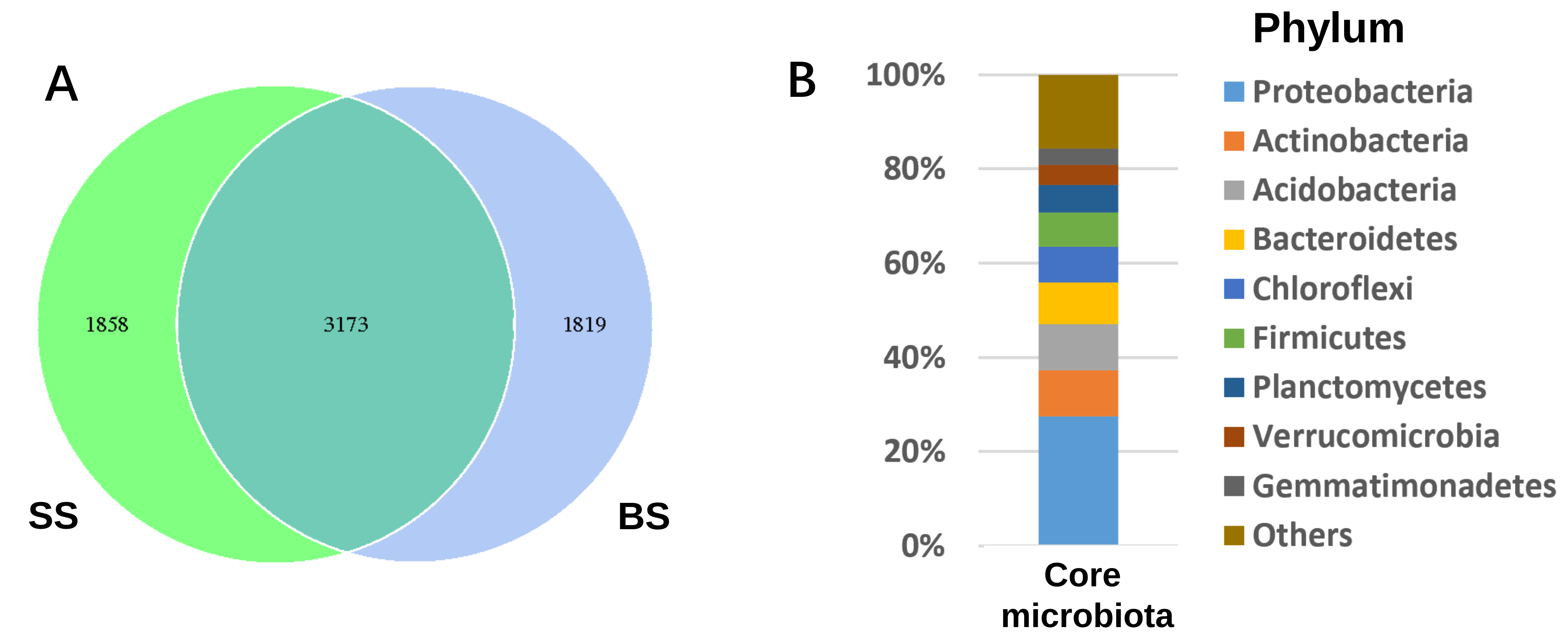

Fig. S2 Identification of shared OTUs of the BS and SS groups.
A. Venn diagram; **B**. Composition of the core microbiota (3173 OTUs) at phylum level.

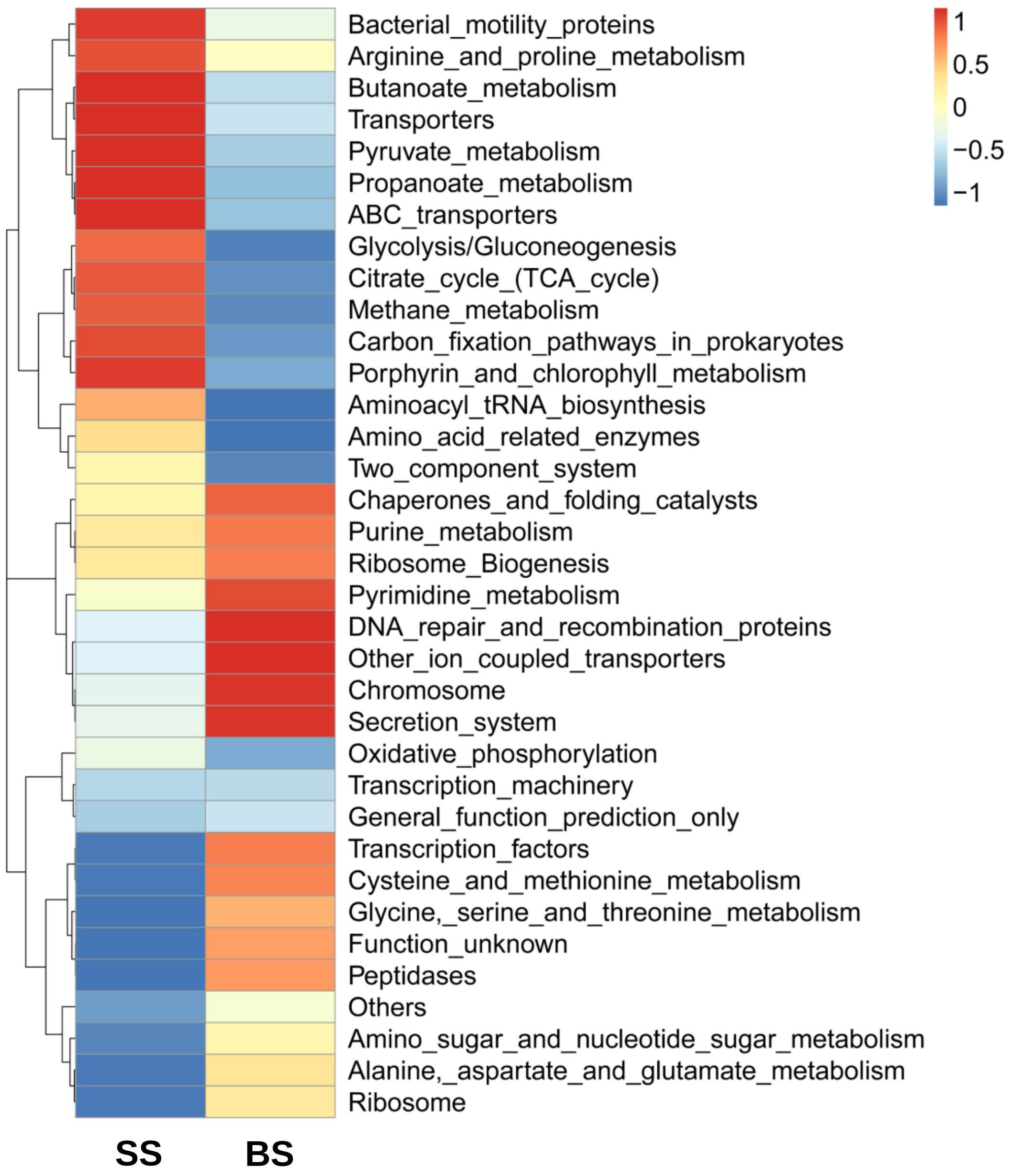

Fig. S3 KEGG function prediction.

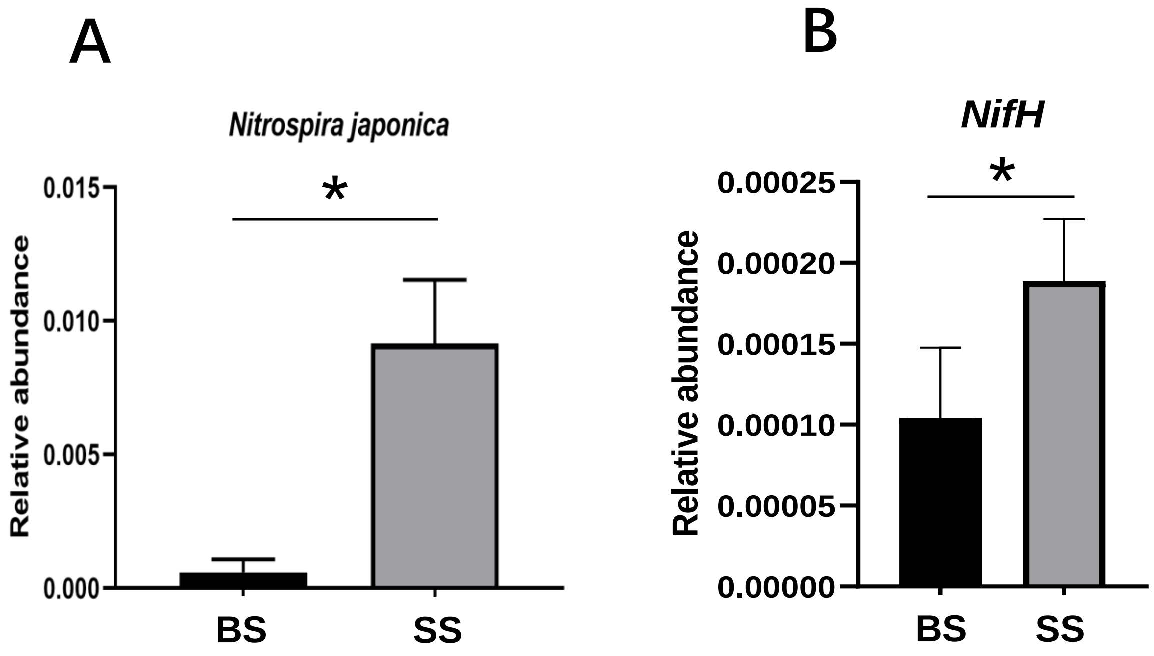

Fig. S4 Relative abundance of nitrogen-fixing bacteria and associated gene.
A. Relative abundance of *Nitrospira japonica* compared between the BS and SS groups.
B. Relative abundance of nitrogen-fixing-associated gene *NifH* compared between the BS and SS groups.

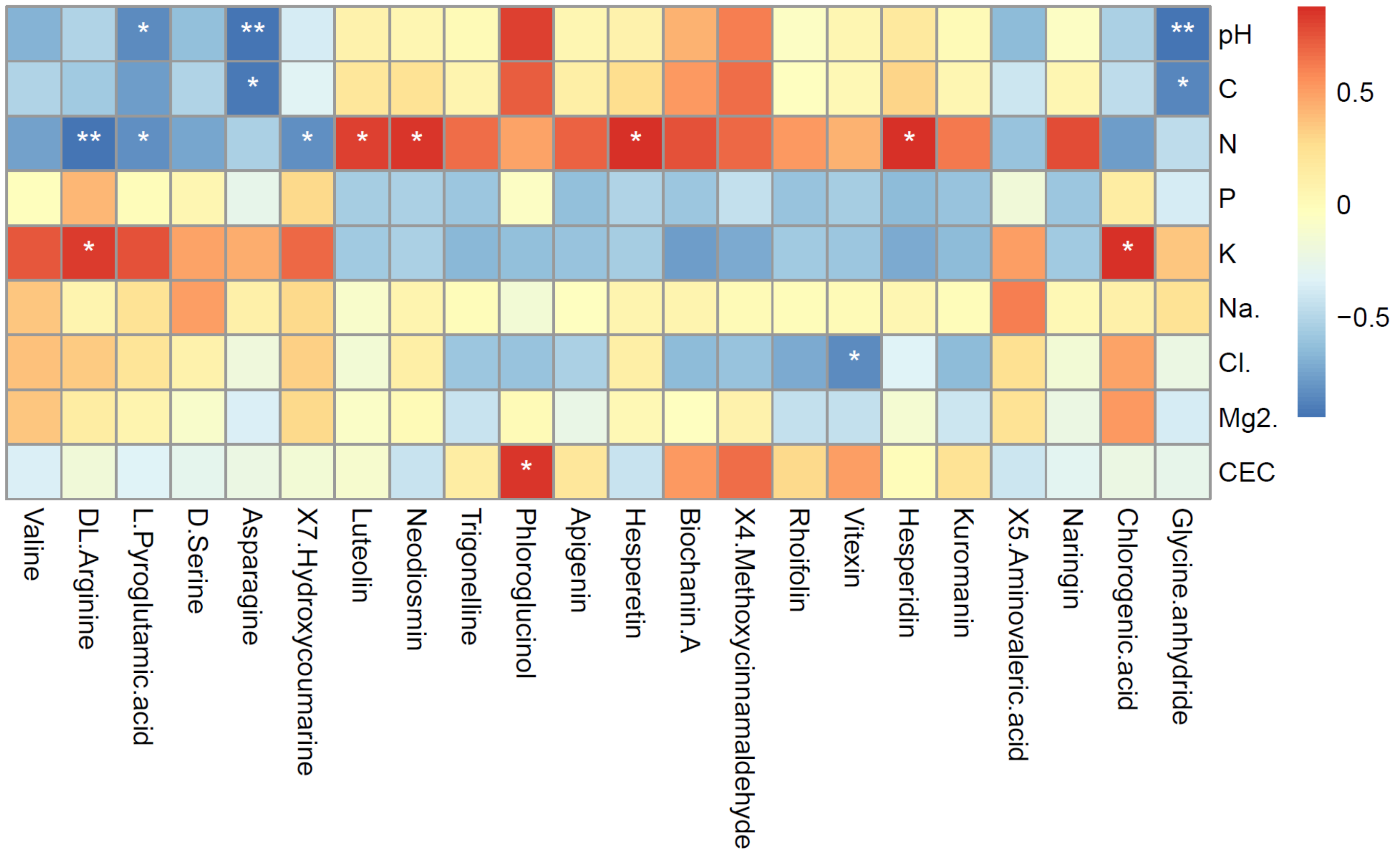

Fig. S5 Correlation analysis between soil properties and distinct metabolites of sugarcane.
(Positive correlation in red, negative correlation in blue; *$p < 0.05$, **$p < 0.01$)
